# Platelet-Monocyte Aggregates and C-Reactive Protein are Associated with VTE in Older Surgical Patients

**DOI:** 10.1038/srep27478

**Published:** 2016-06-07

**Authors:** Lauren Shih, David Kaplan, Larry W. Kraiss, T. Charles Casper, Robert C. Pendleton, Christopher L. Peters, Mark A. Supiano, Guy A. Zimmerman, Andrew S. Weyrich, Matthew T. Rondina

**Affiliations:** 1 Department of Internal Medicine, University of Utah, Salt Lake City, USA; 2 Division of Vascular Surgery, University of Utah, Salt Lake City, USA; 3 Study Design and Biostatistics Center, University of Utah, Salt Lake City, USA; 4 Department of Orthopedic Surgery, University of Utah, Salt Lake City, USA; 5 Division of Geriatrics, University of Utah, Salt Lake City, USA; 6 Molecular Medicine Program at the University of Utah Health Sciences Center, Salt Lake City, Utah; 7 George E. Wahlen Salt Lake City VAMC GRECC, Salt Lake City, USA

## Abstract

Emerging evidence implicates platelets as key mediators of venous thromboembolism (VTE). Nevertheless, the pathways by which platelets and circulating procoagulant proteins synergistically orchestrate VTE remain incompletely understood. We prospectively determined whether activated platelets and systemic procoagulant factors were associated with VTE in 32 older orthopedic surgery patients. Circulating platelet-monocyte aggregates (PMAs), p-selectin expression (P-SEL), and integrin αIIbβ3 activation (PAC-1 binding) were assessed pre-operatively and 24 hours post-operatively. The proinflammatory and procoagulant molecule C-reactive protein (CRP), which induces PMA formation *in vitro*, along with plasma d-dimer and fibrinogen levels were also measured. The primary outcome was VTE occurring within 30 days post-operatively. Overall, 40.6% of patients developed VTE. Patients with VTE had a significant increase in circulating PMAs and CRP post-operatively, compared to those without VTE. Changes in PMA and CRP in VTE patients were significantly correlated (r^2^ = 0.536, p = 0.004). In contrast, P-SEL expression and PAC-1 binding, fibrinogen levels, and d-dimers were not associated with VTE. This is the first study to identify that increased circulating PMAs and CRP levels are early markers associated with post-surgical VTE. Our findings also provide new clinical evidence supporting the interplay between PMAs and CRP in patients with VTE.

Major orthopedic surgery, including total hip and knee arthroplasty (THA/TKA) increases the risk of venous thromboembolism (VTE). Older age is also a VTE risk factor, with an approximately 7–10 fold increased risk in adults less than 55 years old as compared to adults 75 years and older^1^,^2^,^3^. While comorbid conditions contribute to the increased incidence of VTE in older adults, aging-specific factors including platelet reactivity, endothelial dysfunction, and altered hemostatic responses are also thought to influence thrombosis risk but remain incompletely understood^1^,^4^.

Emerging and established evidence implicates platelets as key cellular mediators of inflammation, vascular disease, and VTE^5^,^6^. As platelets are central to thrombin generation and fibrin deposition, and endothelial injury is common in surgical patients, increased platelet activation and interactions with target cells may contribute to post-surgical VTE^5^,^7^. Moreover, indices of platelet activation may serve to identify patients at increased risk of VTE and thus guide more effective measures to prevent thrombosis. One of the most sensitive markers of *in vivo* platelet activation is increased circulating platelet-monocyte aggregates (PMAs). PMAs are associated with adverse clinical outcomes in older septic patients^8^,^9^ and in patients with arterial thrombosis^8^,^10^,^11^, but have not been studied in older adults undergoing orthopedic surgery.

The pro-inflammatory and pro-coagulant molecule C-reactive protein^12^,^13^ (CRP) is tied to an increased risk of cardiovascular disease. Interestingly, in experimental models, CRP promotes PMA formation and platelet adhesion to endothelial cells^14^,^15^,^16^. Nevertheless, studies investigating whether circulating PMAs and CRP may mediate VTE in human thrombotic disease settings remain absent. The goal of this study was to determine whether increased circulating PMA formation and CRP levels are associated with post-operative VTE in older adults undergoing orthopedic surgery.

## Results

### Clinical Features and Thrombotic Events in the Study Cohort

We prospectively studied 32 patients, who had an average age of 65.5 ± 7.1 years (Table 1). There was no clinical evidence of VTE pre-operatively in these patients. Almost all patients (n = 31/32, 96.9%) underwent surgery for degenerative joint disease due to osteoarthritis. VTE was diagnosed in 40.6% (n = 13/32) of patients and most thrombotic events were DVT (Table 2). All VTE events were diagnosed on the initial compression ultrasound (CUS). The second ultrasound done 14 days post-operatively showed in some cases partial thrombus resolution but did not identify any new DVT. One patient presented with symptomatic PE diagnosed by CT pulmonary angiography. Distal DVT was more common than proximal DVT (76.9% vs. 23.1%, p = 0.09). Of patients with distal DVT (n = 10), three had bilateral distal DVT. Of patients with unilateral distal DVT, four were ipsilateral and one was contralateral to the surgical site, and two occurred in the setting of bilateral TKA. There were no differences in age, gender, BMI, surgery type, tourniquet time (for TKA), or baseline laboratory values between patients with and without VTE (Table 1). Post-operative bleeding and infectious complications were uncommon (Table 2). The identification of DVT altered clinical management with extended duration warfarin monotherapy and/or the use of enoxaparin in addition to warfarin in most patients (Table 2). As expected, patients diagnosed with VTE had a higher TTR and a longer duration of anticoagulation than patients without VTE (62.1% vs. 40%, p < 0.05 and 61.0 ± 11 days vs. 17.4 ± 5.0 days, respectively; p < 0.001), highlighting that a diagnosis of VTE altered clinical management.

### Circulating PMAs and CRP were Associated with VTE

**Figure 1:**
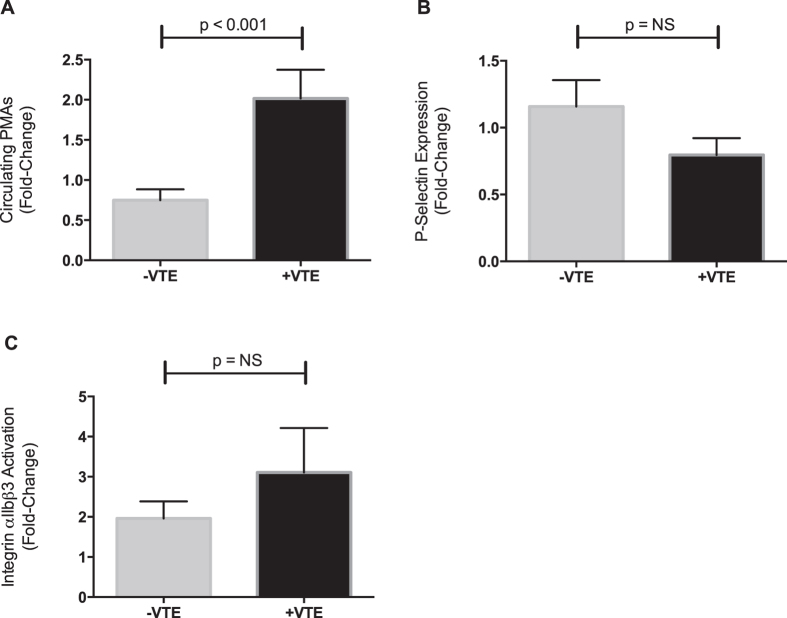
Increased circulating platelet-monocyte aggregates (PMAs) are associated with post-operative VTE. Circulating PMAs, platelet surface P-selectin expression (P-SEL), and integrin αIIbβ3 activation were measured by whole blood flow cytometry immediately prior to surgery and again 24 hours post-operatively. The number of PMAs, the expression of P-SEL, and integrin αIIbβ3 activation post-operatively was compared to pre-operative (or baseline) values and the fold-change was determined for each patient. (**A**) Circulating numbers of PMAs were significantly increased post-operatively in patients who developed VTE (+VTE, n = 13), compared to patients without VTE (−VTE, n = 19). In comparison, neither (**B**) P-SEL expression nor (**C**) integrin αIIbβ3 activation was associated with VTE. Data show the mean ± SEM for each group.

Circulating PMAs were significantly increased in patients with VTE on POD1 whereas those without VTE had unchanged (or slightly reduced) circulating PMAs (Fig. 1A). P-SEL expression was unchanged post-operatively and did not differ significantly between patients with and without VTE (Fig. 1B). Platelet integrin αIIbβ3 activation increased post-operatively but did not significantly differ between patients with and without VTE (Fig. 1C). In pre-specified subgroup analyses excluding patients given aspirin post-operatively (n = 13), post-operative changes in circulating PMA levels remained significantly higher in patients with VTE compared to those without VTE (fold-change of 1.48 ± 0.20 vs. 0.65 ± 0.14, p < 0.01).

**Figure 2:**
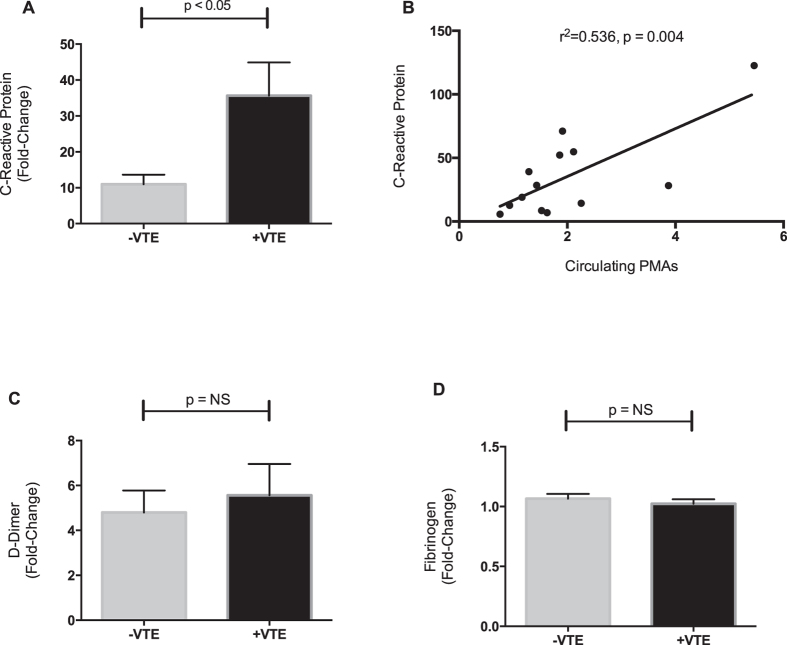
C-reactive protein (CRP) is increased in patients with VTE and correlates with platelet-monocyte aggregates (PMAs). Plasma was harvested from whole blood collected immediately pre-operatively and again 24 hours post-operatively in all patients. Levels of CRP, d-dimer, and fibrinogen were measured. Shown is the fold-change post-operatively compared to pre-operative (e.g. baseline) levels in each patient. (**A**) Changes in CRP were significantly higher in patients who developed VTE (+VTE, n = 13), compared to patients without VTE (−VTE, n = 19) and (**B**) correlated with increased PMAs. In contrast, changes in (**C**) d-dimer and (**D**) fibrinogen did not significant differ between patients who developed VTE and those who did not develop VTE. Data show the mean ± SEM for each group. The Pearson’s coefficient (r^2^) was determined for the correlation between CRP and PMAs.

CRP levels were also significantly increased in patients with VTE (Fig. 2A). Increases in CRP, which has been demonstrated to orchestrate PMA formation *in vitro*^14^, correlated significantly and positively with increased PMAs (Fig. 2B). CRP levels were not correlated with P-SEL expression (r^2^ = 0.04, p = 0.25) or integrin αIIbβ3 activation (r^2^ = 0.005, p = 0.69). Plasma levels of d-dimer rose following surgery, as expected, but were not associated with VTE (Fig. 2C) and did not significantly correlate with PMAs (r^2^ = 0.37, p = 0.08). Plasma fibrinogen levels remained generally unchanged after surgery and did not differ between patients who developed VTE and those who did not (Fig. 2D).

## Discussion

Here we prospectively show for the first time that increased circulating PMAs following orthopedic surgery are associated with VTE in older patients. Moreover, increased CRP was also associated with VTE and significantly correlated with changes in circulating PMAs. Increased levels of both circulating PMAs and plasma CRP have been tied to cardiovascular disease in humans. While still incompletely understood, emerging evidence suggests that CRP may have previously unrecognized pro-thrombotic effects. CRP increases the expression of adhesion molecules and plasminogen-activator inhibitor 1 (PAI-1) in endothelial cells^12^,^17^ and the expression of tissue factor (TF) in monocytes^18^. Transgenic mice infused with human CRP exhibit increased thrombosis and in human volunteers infused with exogenous CRP, there is an increase in pro-thrombotic factors^13^,^19^. CRP induces platelet adhesion to endothelial cells^15^,^16^, one of the initial critical steps for vascular thrombosis. Importantly, CRP also promotes PMA formation *in vitro* and *in vivo* models of transgenic mice. In the REGARDS study, which examined inflammatory markers and incident VTE in 30,239 subjects, higher CRP levels were associated with an increased risk of VTE^20^. Our findings that increases in CRP correlated with PMAs and were associated with VTE in orthopedic surgery, a setting where inflammation and vascular wall damage occur, provides further evidence supporting the link between platelet activation, CRP, and thrombosis.

The results of this investigation extend published studies demonstrating that circulating PMAs are sensitive markers of *in vivo* platelet activation and are consistent with findings in older patients with other acute thrombotic and inflammatory conditions^8^,^9^,^10^. Platelet surface P-selectin expression, which mediates adhesion of platelets to monocytes, was not associated with VTE in our study. This is similar to other studies of P-selectin and thrombosis in orthopedic surgery^21^, perhaps due in part to the rapid shedding of P-selectin from the platelet surface once activated. In addition, once stimulated, platelets may adhere to injured or activated endothelial cells and to growing thrombus. The results of our study are also consistent with previous studies of plasma-based platelet activation markers in surgery. For example, levels of ADAMTS13, von Willebrand factor, platelet factor 4, and CD40 ligand, which are all secreted by platelets when activation, increase post-operatively and, in some settings, correlate with thrombosis risk^22^,^23^. Finally, as PMA formation mediates pro-inflammatory gene synthesis and associated pro-thrombotic responses^5^, these findings support the role of platelets as mediators in the pathogenesis of VTE.

PMA formation in infectious and inflammatory settings is dysregulated in older adults, leading to exaggerated cytokine synthesis and adverse clinical outcomes^9^. Our results build upon these clinical studies in older populations, demonstrating that PMAs are also associated with thrombotic events following orthopedic surgery. As both older age and orthopedic surgery are risk factors for VTE, and measurements of PMAs and CRP in this study were made immediately before and 24 hours after surgery, our findings, may lead to improvements in VTE risk stratification and early diagnosis in this higher risk population, if confirmed in larger studies.

The strengths of this study include the prospective design, our rigorous assessment of *in vivo* platelet activation by three distinct indices, our inclusion of correlative plasma procoagulant markers for comparison, and our comprehensive assessment of VTE, including the use of bilateral, comprehensive duplex venous ultrasonography at two time points postoperatively. While the majority of VTE that occurred in our patients was distal DVT, consistent with published reports in this population^24^, these thrombotic events were clinically significant, resulting in extension of anti-thrombotic therapy. Moreover, approximately one-third of these distal DVTs were bilateral, which are associated with higher rates of recurrence and mortality than unilateral distal DVT^25^,^26^,^27^. Thus, while the management of distal DVT remains controversial, in our older surgical patients in our study, identification of thrombotic events influenced patients’ treatments.

While d-dimer levels increased post-operatively, they were not associated with VTE. Increases in D-dimer are a sensitive, albeit non-specific, diagnostic tool in patients with suspected VTE and helps guide clinical evaluations. Our data suggest that d-dimer may have less utility in the early post-operative period for identifying patients at increased risk of post-surgical VTE. We also identified a relatively high frequency of DVT. Reasons for this are not entirely clear and may be due to the timing of the US relative to surgery or changing demographics in this population and the older age of the cohort. As all patients received guideline-recommended thromboprophylaxis^28^, however, we do not believe that this high incidence reflects suboptimal VTE prophylaxis management. While not a primary focus of this study, our findings also suggest that in selected high-risk patients (perhaps those with evidence of enhanced *in vivo* platelet activation), more aggressive early anticoagulation may be needed. Finally, while prospective in nature and rigorous in design, our study is limited by the small sample size and further studies are needed to further examine platelet activation and VTE in older adults.

## Conclusions

In conclusion, this study identifies that increased circulating numbers of PMAs and CRP levels within 24 hours after major orthopedic surgery are early markers associated with VTE in older adults.

## Methods

### Patient Recruitment

The University of Utah Institutional Review Board (IRB # 26265) approved this study, all patients provided informed consent, and all methods were carried out in accordance with IRB approved guidelines. Adults undergoing elective THA or TKA (American Society of Anesthesiologists Physical Class I or II) were included. Patients with a history of venous or arterial thrombosis, known thrombophilia, uncontrolled hypertension (BP ≥ 160/95 mmHg), chronic kidney or liver disease, or surgery, hospitalization, acute coronary syndrome, or red blood cell or platelet transfusions within 30 days prior to surgery were excluded. No patients received pre-operative anticoagulation. NSAIDS were discontinued ≥7 days pre-operatively in all patients. Patients taking aspirin (81–162 mg daily) pre-operatively were allowed to continue the aspiring if recommended by their physician; otherwise aspirin was stopped ≥7 days prior to surgery.

Demographic and clinical data were prospectively captured for all patients. Routine laboratory data and the procoagulant factors CRP, d-dimer, and fibrinogen concentration were measured immediately prior to surgery and 24 hours post-operatively (e.g. post-operative day 1; POD1) by a national reference laboratory using standard quality controls.

The primary outcome was the development of any VTE, including deep vein thrombosis (DVT) and pulmonary embolism (PE) within 30 days post-operatively. Patients were also followed for any major or clinically relevant non-major (CRNM) bleeding^29^. Major bleeding was defined as fatal bleeding, symptomatic bleeding in a critical organ, extra surgical site bleeding causing a fall in hemoglobin level of ≥2.0 g/L leading to transfusion, surgical site bleeding requiring a second intervention, or unexpected and prolonged surgical site bleeding resulting in hemodynamic instability and transfusion. CRNM bleeding was defined as overt bleeding not meeting the criteria for a major bleeding event but associated with medical intervention, unscheduled contact with a physician, or discomfort or impairment of activities of daily life.

### Platelet Activation Studies

Whole blood was carefully drawn via peripheral venipuncture into sterile acid-citrate-dextrose BD vacutainer tubes®. The first 5mLs of blood were discarded and the remaining blood was immediately transported at room temperature to the laboratory without agitation. Blood samples were drawn between the hours of 7–10AM immediately prior to surgery and at the same time on POD1, simultaneous when clinical laboratory parameters and procoagulant markers were measured. Platelet integrin αIIbβ3 binding, platelet surface p-selectin expression (P-SEL), and circulating numbers of PMAs, indices of *in vivo* platelet activation, were assessed in unstimulated whole blood by flow cytometry within 20 minutes of blood collection as previously described^9^,^30^,^31^. Briefly, whole blood was incubated with the platelet marker CD41 phycoerythrin and the monocyte marker CD14 (for PMAs), the fluorescein isothiocyanate monoclonal antibody for PAC-1 (binds to the active conformation of integrin αIIbβ3), or CD62 (for P-SEL). Samples were analyzed with a FACScan Analyzer and CellQuest software. Antibodies were obtained from BD Biosciences. Integrin αIIbβ3 activation, P-SEL expression, and circulating PMAs were adjusted for non-specific binding. Changes in these indices of *in vivo* platelet activation (defined as the fold-change between pre-operative and POD1 values) were determined for each patient.

### Thromboprophylaxis

Adjusted-dose warfarin monotherapy (target INR 2.0–2.5 per institutional guidelines) was used post-operatively for VTE prophylaxis. Warfarin was initiated the evening after surgery and managed by a team of anticoagulation specialists using a warfarin nomogram^32^. Warfarin management was evaluated by determining time in therapeutic range (TTR)^33^. When VTE was diagnosed, the patient’s medical team made all anticoagulant treatment decisions. The use of parenteral anticoagulants and duration of anticoagulation was prospectively recorded. Intermittent compression devices and compression stockings were used post-operatively in all patients.

### Compression Ultrasonography

Registered vascular technologists performed comprehensive bilateral, lower extremity duplex compression ultrasonography (CUS) on all patients at two time points following surgery. The first CUS was performed prior to hospital discharge and a second CUS was performed approximately 14 days postoperatively, per standardized protocol^34^. Briefly, the following deep veins of the thigh and calf were examined in a sequential manner in 1–2 cm increments: common femoral vein, mid and distal femoral vein, popliteal vein, trifurcation of the deep calf veins, posterior tibial veins, the gastrocnemius veins, soleal sinus vein, and peroneal veins. Lack of venous compressibility with the ultrasound transducer held in a transverse position to the vein was interpreted as a positive study. Absent or diminished color flow and pulse wave doppler with failure of augmentation further confirmed the presence of DVT. A board-certified vascular surgeon, blinded as to the patient’s clinical information, measurements of *in vivo* platelet activation, and procoagulant markers, interpreted the CUS. Proximal DVT was defined as an acute-appearing thrombosis involving the popliteal and/or more proximal lower extremity deep vein segments. Distal DVT was defined as an acute appearing thrombosis in any deep vein segment distal to the popliteal vein.

### Statistical Analyses

Data were examined for normality using skewness and kurtosis tests. Groups were compared using the student’s t-test or Wilcoxon Rank Sum and the Chi-squared or Fisher’s exact test, as appropriate (GraphPad Prism v6.0, La Jolla, CA). Central tendency data are reported as the mean or median (interquartile range, IQR) if the distribution was skewed. Pearson’s regression analyses were performed to analyze correlations. A p-value < 0.05 was considered statistically significant.

## Additional Information

**How to cite this article**: Shih, L. *et al.* Platelet-Monocyte Aggregates and C-Reactive Protein are Associated with VTE in Older Surgical Patients. *Sci. Rep.* **6**, 27478; doi: 10.1038/srep27478 (2016).

## Figures and Tables

**Table 1:** Characteristics of the study cohort and comparisons between patients with and without venous thromboembolism (VTE).

	Overall (n = 32)	No VTE (n = 19)	VTE (n = 13)	*P*value
*Demographics & Clinical Features*				
Age, years	65.6 ± 7.1	65.6 ± 8.0	65.5 ± 5.7	0.95
Male Gender, n (%)	14 (43.8%)	7 (36.8%)	7 (53.8%)	0.91
BMI, kg/m^2^	30.8 ± 6.5	30.4 ± 6.5	31.4 ± 6.7	0.67
Obesity (BMI ≥ 30kg/m^2^), n (%)	17 (53.1%)	8 (47.4%)	8 (61.5%)	0.43
*Baseline Pre-operative Values*				
Hemoglobin, mg/dL	13.6 ± 1.2	13.5 ± 1.3	13.9 ± 1.1	0.29
Platelets, K/uL	282 ± 62	274 ± 52	292 ± 75	0.44
White Blood Cell Count, K/uL	7.4 ± 2.6	7.4 ± 3.1	7.3 ± 1.8	0.99
International Normalized Ratio	1.0 ± 0.1	1.0 ± 0.1	1.0 ± 0.1	0.91
Prothrombin Time, sec	13.1 ± 0.6	13.1 ± 0.7	13.1 ± 0.7	0.92
aPTT, sec	31.4 ± 3.7	31.0 ± 2.4	32.0 ± 5.1	0.45
*Surgical Characteristics*				
Total knee arthroplasty (TKA), n (%)	23 (71.9%)	13 (68.4%)	10 (76.9%)	0.70
Unilateral TKA, n (%)	20 (62.5%)	13 (68.4%)	7 (53.8%)	0.07
Bilateral TKA, n (%)	3 (9.4%)	0 (0%)	3 (100%)	0.07
Total hip arthroplasty (THA), n (%)	9 (29.1%)	6 (31.6%)	3 (23.1%)	0.70
Tourniquet time (for TKA), min.	56.1 ± 12.6	55.5 ± 16.0	56.9 ± 7.0	0.80

Central tendency data are reported as mean (±SD) unless otherwise specified (BMI: body mass index; aPTT: activated partial thromboplastin time).

**Table 2:** Anticoagulation management and clinical outcomes of the study cohort.

*Anticoagulation Management*	Value
Duration of OAC, days (median [IQR])	19 (13–29)
Overall cTTR, (%)	51%
Days to INR > 1.9 (median [IQR])	5 (4–8)
Post Operative Aspirin Use, n (%)	4 (12.9%)
*Thrombotic Outcomes*^*¥*^	
Any VTE, n (%)	13 (41.9%)
Proximal DVT, n (%)	3 (9.6%)
Unilateral Distal DVT, n (%)	7 (22.6%)
Bilateral Distal DVT, n (%)	3 (9.6%)
Pulmonary Embolism, n (%)	1 (3.2%)
*Bleeding and Infection Outcomes*	
Major Bleeding, n (%)	0 (0%)
Clinically-Relevant Non-Major Bleeding, n (%)	1 (3.2%)
Infection, n (%)	2 (6.4%)
*VTE Treatment*	
Extended Duration of Warfarin Monotherapy, n (%)	3 (23.1%)
Enoxaparin plus Warfarin, n (%)	8 (61.5%)
Standard Duration of Warfarin Monotherapy, n (%)	2 (15.4%)

Central tendency data are reported as mean ± SD unless otherwise specified (cTTR: center-specific time in therapeutic range; INR: international normalized ratio; OAC: oral anticoagulation; ^¥^patients may have had DVT with or without pulmonary embolism and thus the total number of events may exceed 13).
